# Wide mutation spectrum and frequent variant Ala27Thr of *FBN1* identified in a large cohort of Chinese patients with sporadic TAAD

**DOI:** 10.1038/srep13115

**Published:** 2015-08-14

**Authors:** Jun Guo, Lun Cai, Lixin Jia, Xiaoyan Li, Xin Xi, Shuai Zheng, Xuxia Liu, Chunmei Piao, Tingting Liu, Zhongsheng Sun, Tao Cai, Jie Du

**Affiliations:** 1 Beijing Anzhen Hospital, Capital Medical University, The Key Laboratory of Remodeling-Related Cardiovascular Diseases, Ministry of Education, Beijing Collaborative Innovation Center for Cardiovascular Disorders, Beijing Institute of Heart, Lung & Blood Vessel Disease, Beijing, China,; 2 The Institute of Genomic Medicine, Wenzhou Medical College, Wenzhou, China

## Abstract

Genetic etiology in majority of patients with sporadic thoracic aortic aneurysm and dissections (STAAD) remains unknown. Recent GWAS study suggested common variant(s) in *FBN1* is associated with STAAD. The present study aims to test this hypothesis and to identify mutation spectrum by targeted exome sequencing of the *FBN1* gene in 146 unrelated patients with STAAD. Totally, 15.75% of *FBN1* variants in STAAD were identified, including 5 disruptive and 18 missense mutations. Most of the variants were novel. Genotype-phenotype correlation analysis suggested that the maximum aortic diameter in the disruptive mutation group was significantly larger than that in the non-Cys missense mutation group. Interestingly, the variant Ala27Thr at −1 position, which is predicted to change the cleavage site of the signal peptidase of fibrillin-1, was detected in two unrelated patients. Furthermore, genotyping analysis of this variant detected 10 heterozygous Ala27Thr from additional 666 unrelated patients (1.50%), versus 7 from 1500 controls (0.47%), indicating a significant association of this variant with STAAD. Collectively, the identification of the variant Ala27Thr may represent a relatively common genetic predisposition and a novel pathogenetic mechanism for STAAD. Also, expansion of the mutation spectrum in *FBN1* will be helpful in genetic counselling for Chinese patients with STAAD.

Thoracic aortic aneurysm and dissection (TAAD) is a frequent cause of morbidity and mortality in patients with cardiovascular disorders. Thoracic aortic aneurysms are often asymptomatic and underdiagnosed and may lead to an acute aortic dissection with life-threatening complications. Many presentations of TAAD show classic Mendelian inheritance with high or complete penetrance, suggesting the major contribution of a single gene^1^. The most common genes mutated in familial TAAD encode proteins involved in transforming growth factor-beta (TGF-β) signaling (*TGFBR1/2*^2^,^3^, *TGFB2*^4^, *SKI*^5^, and *SMAD3*^6^) or proteins that comprise the contractile apparatus of aortic smooth muscle cells (*MYH11*^7^, *ACTA2*^8^, and *MYLK*^9^). Mutations of *FBN1* were more frequently detected in patients with Marfan syndrome (MFS) including many Chinese patients^10^,^11^ compared to that in patients with STAAD^12^,^13^,^14^.

However, in the absence of familial history and features of syndromic forms, the proportion of patients with sporadic TAAD (STAAD) resulting from a genetic predisposition remains unknown. A recent GWAS study^15^ has mapped a single susceptibility locus of STAAD to 15q21.1, suggesting that one or more common variants within the *FBN1* gene are associated with STAAD. This hypothesis whether *FBN1* common variants that may affect the gene’s function and expression can be identified in affected individuals with STAAD needs to be tested by large-sample sequencing studies.

With the progress in next-generation sequencing (NGS), targeted NGS of disease-specific genes can be reliably implemented as a stand-alone diagnostic test. Recent studies by us and others^16^,^17^ have demonstrated this technology has higher efficiency but lower cost compared with traditional methods. Here, we report the results from 146 STAAD patients by target NGS analysis and additional 666 STAAD patients by mutation association study.

## Results

### Patient cohort

A total of 146 patients (115 males and 31 females) with a mean age of 50.31 years (16–81 years) were consecutively recruited in our hospital. Half of the patients were younger than 50 years old when the diagnosis was first given. Only 10 of the individuals were under the age of 31 years. Aortic dissections were found in 92 (63.01%) cases, while thoracic aortic aneurysms (TAA) were diagnosed in 54 (36.99%). Additional 8 individuals were classified into MFS^18^,^19^, because of accompanying skeletal and/or ocular complications (such as arachnodactyly, scoliosis, pectus carinatum, and severe myopia), and thus were excluded from this study. No patient was found with Shprintzen-Goldberg syndrome^5^ or a newly defined aortic aneurysm syndrome due to TGFβ receptor mutations^3^.

### Identification of *FBN1* variants

In order to identify the mutation spectrum of *FBN1* in Chinese STAAD, we sequenced each of the 146 DNA samples using the NGS technology. An average sequencing depth of these samples in the targeted regions reached 53.07× (ranging from 43.61× to 74.21×). Each sample had more than 81.1% of the targeted regions being covered. An average coverage of targeted exons (>10 reads) was 75.0%, and also the coverage (>20 reads) reached 74.0%.

**Figure 1:**
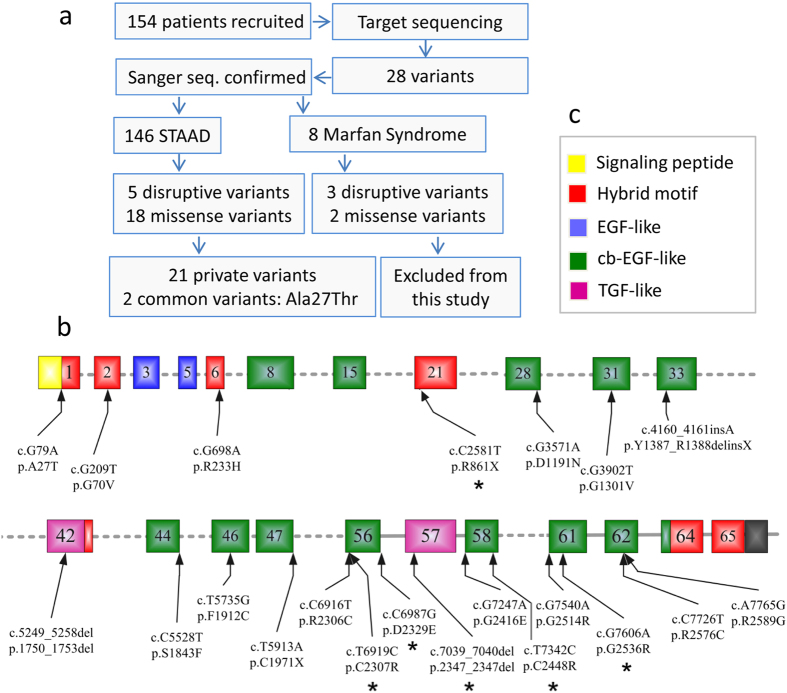
Research steps and identification of *FBN1* variants. (**a**) The scheme illustrates the main steps of patient classification and targeted sequencing results. (**b**) Schematic representation of locations of 23 *FBN1* variants. Previously reported variants are labeled by stars*. (**c**) Symbol keys: Selected exons (numbered in each box) encoding for signal peptide are in yellow, for hybrid motifs in red, for epidermal growth factor (EGF)-like domains in blue, for calcium-binding epidermal growth factor (cb EGF)-like domains in green, and for transforming growth factor beta (TGF-β)-like domains in purple.

A total of 23 *FBN1* variants in 23 unrelated patients (15.75%) were identified from the cohort of 146 individuals (Fig. 1a). Average sequencing depth in these 23 patients achieved 52-fold in 80.55% of the target regions (Table S1), which is similar to the sequencing depth reached in the 146 subjects. All detected variants were bidirectionally confirmed by Sanger sequencing (Fig. S1). All variants were found to be present in highly conserved nucleotides of the coding region (Fig. S2-S3), and were mostly predicted to be damaging by SIFT, PolyPhen-2 and MutationTaster. Notably, 14/23 of variants were first identified, not shown in the commonly used databases (ESP6500, 1000G, dbSNP132 and 1500 Chinese Han in-house database). The rest 9/23 variants (Table 1) were rare with low MAF (0.0003–0.0077).

Among the 23 variant carriers detected from the 146 STAAD samples (15.75%), further analysis stratified by phenotype divided 14.81% (8/54) variants to the TAA group and 16.30% (15/92) to the aortic dissections group (12 type A, 3 type B). Only 2 of them were younger than 31 years.

### Recurrent *FBN1* variants

**Figure 2:**
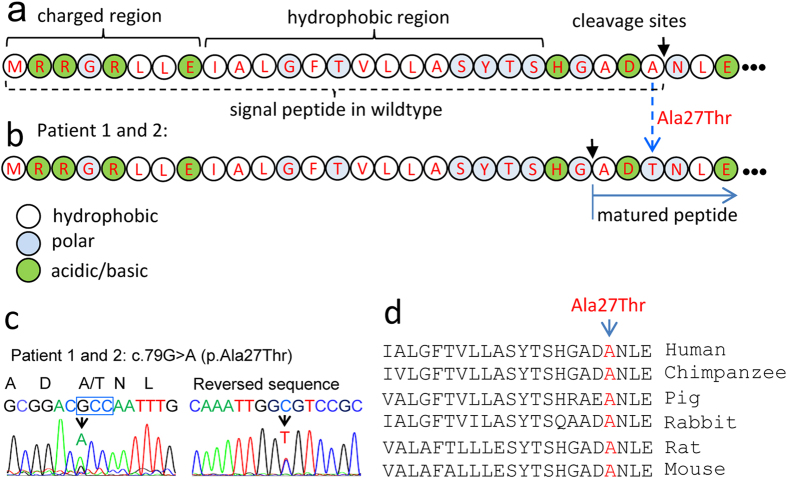
Effect of Ala27Thr on Signal peptide. (**a**) Analysis of signal peptide structure and its amino acid properties. The signal peptide in secreted proteins is comprised of a charged region, a central hydrophobic region that is usually flanked by charged residue and a C-terminal region containing signal peptidase cleavage site(s). The cleavage site (arrow) between Ala27 and Asn28 is predicted by SignalP 4.1. These features are critical for the translocation of secretory proteins and their cleavage by signal peptidase. (**b**) Effect of Ala27Thr on the cleavage site (arrow) in two patients with STAAD. (**c**) The c.79G>A variant is confirmed in Patient 1 and 2 by bidirectional Sanger sequencing. (**d**) Evolutionary analysis of p.Ala27Thr in selected species by multiple sequence alignment.

Of the nine rare SNP variants, p.Ala27Thr was detected in two unrelated patients (Table 1). Ala27 is at the −1 position of the signal peptidase cleavage site (Fig. 2a), as predicted by SignalP 4.1 Server. The substitution of Ala27 with tyrosine (Fig. 2b) was predicted to change the cleavage site of fibirillin-1 (Fig. S4). This substitution was confirmed by Sanger sequencing (Fig. 2c). Ala27 is evolutionarily conserved in most species (Fig. 2d), but not in few others like sheep. And yet, this variant is present in 1000G database (rs25397) with low MAF (=0.0018, given a rare variant is defined by fewer than one in 1,000).

In order to investigate if the c.79G>A (p.Ala27Thr) variant is associated with the disease in a larger cohort of STAAD patients, we performed genotyping analysis using a TaqMan assay in additional 666 unrelated subjects with STAAD. We identified the heterozygous Ala27Thr substitute in 10 of them (1.50%). These substitutions were all confirmed by Sanger sequencing. On the contrary, only 7 were found from 1500 Chinese Han individuals from our in-house database (0.47%), indicating that the c.79G>A is significantly associated with STAAD (χ^2^ test, *P* = 0.012, Table 2).

### Mutation spectrum and genotype–phenotype correlation

Further analysis disclosed that 17 of these variants were novel, and only 6 of them were previously reported in MFS, including, p.Arg861×^20^,^21^, p.Cys2307Arg (*FBN1* mutations database), p.Asp2329Glu^22^, p.Met2347delAT^23^, p.Cys2448Arg^24^, and p.Gly2536Arg^25^. No different clinical manifestations were found between the reported patients and our cases under the same mutations (Table S2).

**Figure 3:**
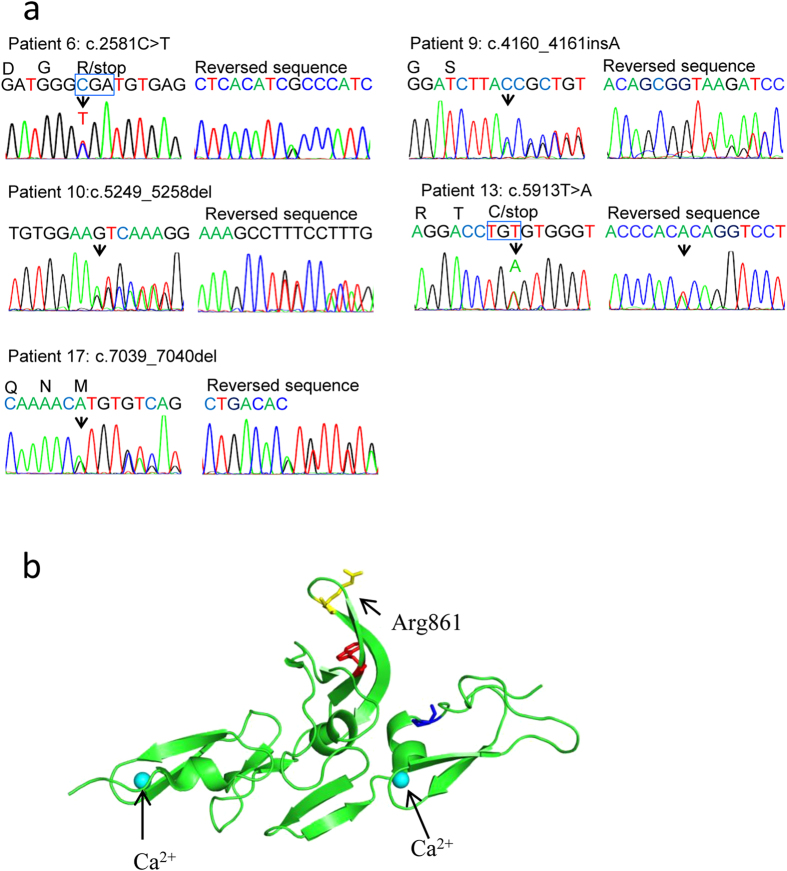
Disruptive mutations and protein structure. (**a**) Sanger sequencing chromatograms of five disruptive mutations identified in five patients with STAAD. (**b**) Structural analysis of fibrillin-1 with a premature stop mutation in the cb EGF-like domain. Locations of residues R861 are shown in yellow. Two calcium binding sites are shown in cyan. 3D structural model is established using PyMOL Molecular Graphics System (Schrödinger L. version 1.3r1, 2010). This structure contains 147 amino acids (residues 807–951) (ID# 2W86, PDB Bank, http://www.pdb.org).

Remarkably, we identified a total of 5 variants (c.2581C>T, c.4160_4161insA, c.5249_5258del GTCAAAGGCC, c.5913T>A and c.7039_7040delAT) that lead to nonsense or frameshift-indel mutations, which were predicted to disrupt the function of fibrillin-1 (Fig. 3a). Three of the disruptive mutations (in bold, Table 1), to the best of our knowledge, were identified for the first time. Based upon available three-dimensional structural data of fibrillin-1, we were able to predict the effect of a premature stop mutation thereof on the function of the protein. Clearly, the variant p.Arg861X was located in the hybrid motif 2, and could result in the loss of calcium-binding activity and disrupt the protein’s function (Fig. 3b).

Furthermore, we evaluated potential correlations between different types of mutations and clinical conditions. No clinical difference was found between the group with missense mutations involving Cys residues in the calcium-binding epidermal growth factor (cb EGF)-like modules and the group with missense in the non-Cys residues (Table 3). However, we found that the maximum aortic diameter (70.8 ± 18.5 mm) in the disruptive mutation group was significantly larger than that in the groups with non-Cys missense mutation (53.9 ± 12.6 mm, *P* = 0.044). Likewise, the maximum aortic diameter in the disruptive mutation group was also larger than that in the groups with Cys missense. However, this difference did not reach statistical significance.

### Clinical analysis in patients with or without *FBN1* mutations

To find whether the patients with *FBN1* mutations had different clinical manifestations from the patients without, we analyzed their clinical characteristics. No differences were found between these two groups in following six cardiovascular system-related parameters, including maximum aortic diameter, incidence of aortic dissection, aortic regurgitation, mitral valve regurgitation, tricuspid valve regurgitation, and bicuspid aortic valve (Table 4). However, the patients with *FBN1* mutations were 6.7 years younger than the patients without *FBN1* mutation (*P* = 0.028) when the first diagnosis of TAAD was given. In addition, 30.4% of the patients with *FBN1* mutation had a clinical history of hypertension vs. 62.6% of the patients without (*P* = 0.006).

## Discussion

In the present study, we have reported the results of *FBN1* mutations in 146 patients with STAAD as well as a rare SNP association study with 666 additional patients. This screening, the largest to date in Chinese cohort, has identified a total of 23 variants, including 17 novel variants, 5 disruptive mutations, and 2 recurrent variants. Majority variants are rare and lead to missense, nonsense and frameshift-indel mutations. Interestingly, the variant (p.Ala27Thr) of *FBN1* is a relatively frequent SNP, which is significantly associated with the disease.

Bioinformatics analyses have shown that most, if not all, of these variants are pathogenic mutations. We have shown that the *FBN1*-mutation rate is approximately 15.75% in 146 Chinese patients with STAAD, compared to 62.5% in the 8 patients with MFS (Fig. 1; Table S3). The rate of *FBN1*-variant was much higher in patients with MFS, which is in agreement with previous studies^26^. However, the incidence of *FBN1*-mutation may show substantial differences in different subjects and different sample sizes. Also, current Ghent nosology for the clinical diagnosis of MFS, given no family history of the disease, include aortic root enlargement plus one of either ectopia lentis or pathogenic *FBN1* mutation or a systemic core greater than or equal to seven^19^. Therefore, all of these 28 patients could be classified into MFS given the pathological effect of each *FBN1* variant is ascertained. By this definition, the overall molecular diagnostic yield for MFS achieved 18.18% (28/154 patients) in our cohort. This mutation rate is similar to previous report (18.42%, 14/76) in patients who fulfill the clinical criteria for diagnosis making of MFS^27^.

Although these 23 *FBN1* variants seemed not to be clustered as a “hot-spot”, most variants either affect highly conserved sequences and/or are predicted to be deleterious. Although more than 1,000 different *FBN1* mutations have been documented^28^,^29^ since the first mutation of the gene was discovered in 1991^30^, most mutations identified here are novel, suggesting highly diversified genetic resources in Chinese patients. A majority of the mutations (15/23) occurred within the cb EGF-like domains of the protein fibrillin-1, which are required for protecting fibrillin-1 from the activity of proteases^31^,^32^. On the other hand, mutations that directly disrupt the calcium-binding consensus sequence or result in perturbation of protein conformation (e.g., cysteine substitutions) could give rise to a disordered interbedded region and thus increase the susceptibility of the monomer to proteolysis^33^.

A comparison between the type or position of each mutation and the patient´s phenotype revealed a potential relation. It appeared that patients with disruptive mutations were diagnosed at younger age. And yet, patients with *FBN1* mutation were 6.7 years younger than patients without *FBN1* mutation (Table 4). Indeed, synthesis of normal-sized fibrillin-1 protein in the disruptive mutation samples was only about 50% of the control’s level^20^. Intriguingly, a history of hypertension was significantly more prevalent in patients without *FBN1* mutation. Herein, it is inferred that hypertension is not a primary element involving the primary pathogenesis of STAAD with *FBN1* mutations.

The p.Ala27Thr variant is predicted to eliminate the cleavage site at Ala27/Asn28 of the signal peptide and thus to produce a shorter signal peptide (1–24) rather than normally 27 residues. This might affect maturation and cellular function of fibrillin-1, suggesting a novel causative mechanism of the disease. In the *N*’-fibrillin overexpressed SF9 and CHO-K1 cells, two cleavage sites actually were observed at Gly24/Ala25 and Ala27/Asn28 by amino-terminal sequencing of secreted fibrillin-1^34^. However, cleavage site(s) of the endogenous protein as well as the mutant p.Ala27Thr in human aortic artery cells remains to be determined.

Sequence variations/mutations of a signal peptide have been identified in multiple human disorders^35^,^36^,^37^. Most signal peptide mutations were found in the hydrophobic regions that lead to impaired protein-protein interaction and defective intracellular translocation^38^,^39^. In relatively rare cases, mutations can be found to affect the −1 or −3 residues at the signal peptidase cleavage site. For instance, p.Ala25Thr mutation at the −1 position of the ADAMTS10 signal peptide significantly diminished secretion of the protein into extracellular region and thus caused Weill-Marchesani syndrome^36^, a rare inherited disorder with generalized mesodermal dysplasia. Likewise, an Ala to Thr substitution at −1 position of the signal peptide in preprovasopressin led to failure of signal peptide processing and reduced secretion and resulted in familial central diabetes insipidus^40^.

The Ala27Thr is a recurrent variant (1.37% or 2/146 patients). Although Ala27Thr is also present in 1000G database (low MAF = 0.0018), a large sample screening in 666 unrelated patients confirmed that this variant is significantly associated with the disease, arguing that this variant is a susceptibility allele for STAAD. This assumption is also supported by the GWAS results, i.e., the *FBN1*-containing region was the only locus linked to STAAD^15^. The significantly linked SNPs, such as rs2118181 and rs1036477 in the *FBN1* region, may be served as biomarkers for identifying patients at risk for STAAD. However, whether these biomarkers are functionally related to the disease is difficult to be verified. Therefore, targeted-exome sequencing of the linked region in large samples is an ideal approach to quickly identify potential variants that may affect the gene’s function.

Alternatively, the Ala27Thr variant might be a recessive allele with reduced expression of *FBN1* in STAAD, suggesting a threshold model that requires a combination of mild genetic defects to trigger clinical manifestation. Although this hypothesis has not been experimentally demonstrated, there is a convincing precedent in a familiar TAAD with recessive mutations in the first aspartic acid of a cb EGF-like domain of *FBN-1*^41^.

Furthermore, similar cases with mutations in single peptide regions in several other diseases have been previously reported. For example, a common polymorphism, either Ala17 or Thr17 in the signal peptide of CTLA-4 T-cell receptor, resulted in decreased cell surface presentation, which was associated with a higher risk of autoimmune disorders^42^. Therefore, our findings support a model in which the subtle effects on signal peptidase cleavage by Ala27Thr may predispose individuals to STAAD.

It is worthy to mention that we also identified a disruptive nonsense mutation p.Arg861X in the hybrid motif 2 domain. Only a few other missense mutations in the hybrid motif 1 or 2 domain were previously described^27^,^43^. Interactions between the residues within the hybrid motif 1 and LTBP-1 and LTBP-4 were previously observed^44^, indicating the hybrid motif involves TGF pathway. However, the underlying pathogenic mechanism remains unclear.

These findings collectively highlight the importance of these correlations in anticipating the clinical consequences of specific *FBN1* mutations. Furthermore, it is worthy to apply whole-exome sequence to identify potential genetic defects in the rest 126 patients who do not carry *FBN1* mutations.

Taken together, we have determined that mutations in *FBN1* are responsible for approximately 15.75% (23/146) of Chinese patients with STAAD who did not fulfil the clinical criteria of MFS. A total of 17 novel mutations, particularly those located in the signal peptide or the hybrid motif regions, may involve different pathogenetic mechanisms. To the best of our knowledge, the Ala27Thr may represent a relatively frequent variant that predisposes individuals to STAAD. Our findings will be helpful in genetic counselling for patients with STADD or MFS as well as their at-risk family members.

## Methods

### Patient population

A total of 146 unrelated Chinese Han patients with STAAD, who were evaluated at Beijing Anzhen Hospital and referred for surgery repair, were recruited from the year 2011 to 2012. Eight patients who showed skeletal and ocular complications were not accounted as STAAD but were included for sequencing analysis. Additional 666 unrelated patient samples recently recruited through Beijing Anzhen hospital were used for SNP genotyping association study. No patients had a family history of TAAD or evidence of a syndromic form of TAAD. Patients with aortic lesions associated with trauma, infection, or aortitis were excluded. Written informed consent was obtained from each of the subjects according to the research protocol approved by the Ethical Review Board of Beijing Anzhen Hospital. The methods were carried out in accordance with the approved guidelines.

Echocardiographic examination was performed for assessment of the aortic aneurysm and dissection. Aortic computed tomography scan was also applied as deemed necessary by treating physicians. Individuals were diagnosed as TAAD if they had a thoracic aneurysm (diameter ≥ 3.6 cm) or dissection of the thoracic aorta. Diagnosis and conditions of type A or B aortic dissections were assessed according to the classification of International Registry of Aortic Dissection (IRAD)^45^,^46^. Aortic, tricuspid valve and mitral valve regurgitations, as well as associated congenital disorders (bicuspid aortic valve, atrial septal defect, and patent ductus arteriosus) were systematically screened. All patients were also examined for musculoskeletal, pulmonary, dermatologic, and ophthalmic phenotypes in MFS based on recent Ghent criteria^19^.

### Disease-related gene enrichment and sequencing

DNA was extracted from whole blood with Blood DNA Purification Kit (Promega, Beijing branch, China). Purity of the DNA samples was assessed with a NanoDrop2000 spectrophotometer (Thermo Scientific, Wilmington, DE, USA). *FBN1* was screened by a GenCap custom enrichment kit (MyGenostics, Beijing, China) as described previously^47^,^48^. Biotinylated single-strand capture probes were designed to tile along the exonic non-repeated regions and intron-exon boundaries of the gene (Table S4). Capture experiment was conducted according to the manufacturer’s protocol. The *FBN1* gene enriched libraries were sequenced by Illumina HiSeq 2000 sequencer, which produced paired reads around 90-bp for further bioinformatics analysis.

### Bioinformatics analysis

Bioinformatics analysis began with the raw sequencing data generated from the Illumina pipeline. After the adapter and low-quality reads were discarded, clean reads were aligned to reference *FBN1* sequence (Hg19) by Burrows-Wheeler Aligner (BWA)^49^. Final BAM files were used for variant calling. Single nucleotide polymorphisms (SNPs) and small insertion/deletions (InDels) were detected by SOAPsnp^50^ and SAMtools^51^, respectively. Variants were annotated by ANNOVAR^52^. Quality Control (QC) was performed at each step of the analysis pipeline.

### Validation and evaluation of variants

All identified rare variants in *FBN1* were bidirectionally sequenced by ABI 3130XL sequencer (Applied Biosystems, Foster City, CA). Variant frequency was compared with 4 major SNP databases: ESP6500 (http://evs.gs.washington.edu/EVS/), 1000G (http://www.1000genomes.org/), dbSNP132 (http://www.ncbi.nlm.nih.gov/projects/SNP/) and 1500 Chinese Han in-house database. Deleterious effect of each variant was assessed by various algorithms, such as SIFT (http://sift.bii.a-star.edu.sg/), PolyPhen-2 (http://genetics.bwh.harvard.edu/pph2/) and MutationTaster (http://www.mutationtaster.org/). Signal peptide analysis was evaluated by SignalP 4.1 Server (http://www.cbs.dtu.dk/services/SignalP/).

### SNP Genotyping

Genotyping of 666 patients was performed using TaqMan platform in 384-well plates and read with the Sequence Detection Software on ABI Prism 7900 instrument (Applied Biosystems, Foster City, CA). Primers and probes specific for the variant c.79G>A were supplied by Applied Biosystems. PCR conditions were: 95 °C for 5 min, followed by 8 cycles (0.5 °C touchdown) for 10 s at 95 °C, 25 s at 64 °C and 20 s at 72 °C, and then followed by 32 cycles of 10 s at 95 °C, 25 s at 60 °C and 20 s at 72 °C. 1500 controls’ data were obtained from 1500 Chinese Han in-house database.

### Statistical analysis

Most data were presented as mean ± SD. Qualitative variables were compared by χ^2^ test and Fisher test. Quantitative variables were analyzed by Students *t* test and one-way ANOVA with Bonferroni post-test. All statistical tests were two-sided, and a *P* value of ≤0.05 was considered significant. SPSS (version 19, IBM, Armonk, NY, USA) was used for all statistical analyses.

## Additional Information

**How to cite this article**: Guo, J. *et al.* Wide mutation spectrum and frequent variant Ala27Thr of *FBN1* identified in a large cohort of Chinese patients with sporadic TAAD. *Sci. Rep.* **5**, 13115; doi: 10.1038/srep13115 (2015).

## Supplementary Material

Supplementary Information

## Figures and Tables

**Table 1:** *FBN1* mutation analysis in 23 unrelated patients with STAAD.

**#**	**Sex**	**Age(y)**	**Nucleotide change**	**Proteinchange**	**Domain**	**Clinical finding**	**SNP ID**	**MAF**
1	M	62	c.79G>A	p.Ala27Thr	signal peptide	TAA, AR	rs25397	0.0018*
								0.0023***
2	M	43	c.79G>A	p.Ala27Thr	signal peptide	Dissec, AR, TVR, MVR	rs25397	0.0018*
								0.0023***
3	F	37	c.209G>T	p.Gly70Val	EGF-CA	Dissec, TVR		—
4	M	45	c.698G>A	p.Arg233His	hybrid motif 1	Dissec, TVR, AR		—
5	M	52	c.698G>A	p.Arg233His	hybrid motif 1	Dissec		—
6	M	35	c.2581C>T	p.Arg861X	hybrid motif 2	TAA, AR, MVR, TVR	rs140583	unknown
7	M	64	c.3571G>A	p.Asp1191Asn	cb EGF-like 14	TAA, AR, MVR	rs370121450	0.0077**
8	F	48	c.3902G>T	p.Gly1301Val	cb EGF-like 17	Dissec		—
9	M	32	**c.4160_4161insA**	**p.Tyr1387_Arg1388delinsX**	cb EGF-like 19	Dissec, AR, MVR, TVR		—
10	M	32	**c.5249_5258delGTCAAAGGCC**	**p.Ser1750delGTCAAAGGCC**	TGF-β like 5	Dissec		—
11	F	61	c.5528C>T	p.Ser1843Phe	cb EGF-like 26	TAA, AR		0.0003***
12	F	29	c.5735T>G	p.Phe1912Cys	cb EGF-like 28	TAA, AR, TVR, MVR		—
13	M	41	**c.5913T**>**A**	**p.Cys1971X**	cb EGF-like 29	Dissec, AR		—
14	M	49	c.6916C>T	p.Arg2306Cys	cb EGF-like 36	Dissec, AR, MVR, TVR		0.0003***
15	M	36	c.6919T>C	p.Cys2307Arg	cb EGF-like 36	Dissec, BAV		
16	F	58	c.6987C>G	p.Asp2329Glu	cb EGF-like 36	TAA	rs363831	0.001*
								0.0004**
17	M	39	c.7039_7040delAT	p.Met2347delAT	TGF-β like 7	Dissec		—
18	F	44	c.7247G>A	p.Gly2416Glu	cb EGF-like 37	TAA, AR, TVR		—
19	M	59	c.7342T>C	p.Cys2448Arg	cb EGF-like 38	Dissec, AR		—
20	M	57	c.7540G>A	p.Gly2514Arg	cb EGF-like 39	TAA, AR, MVR, TVR	rs363811	unknown
21	M	22	c.7606G>A	p.Gly2536Arg	cb EGF-like 40	Dissec, AR, TVR		—
22	F	47	c.7726C>T	p.Arg2576Cys	cb EGF-like 41	TAA, AR		—
23	F	40	c.7765A>G	p.Arg2589Gly	cb EGF-like 41	Dissec, AR	rs111413134	unknown

Note: TAA, thoracic aortic aneurysms; Dissec, thoracic aortic dissection; AR, aortic regurgitation; MVR, mitral valve regurgitation; TVR, tricuspid valve regurgitation; BAV, bicuspid aortic valve. MAF (minor allele frequency): *1000G; **ESp6500; ***1500 Chinese Han in-house. -, absent.

**Table 2:** Association between the variant c.79G>A (Ala27Thr) and STAAD.

**SNP**	**Chr.**	**Position (bp)**	**Allele**	**Cases (%)**	**Controls (%)**	***P-value***
c.79G>A	15	48936888	G/A	666 (1.50)	1500 (0.47)	0.012

Data in this table were analyzed by Chi-square test.

**Table 3:** Analysis of clinical conditions in patients with different *FBN1* genotypes.

**Mutation Type**	**Age (yr) at diagnosis**	**Maximum aortic diameter (mm)**	**Aortic dissection (n=)**	**AR (n=)**	**MVR (n=)**	**TVR (n=)**	**BAV (n=)**
Cys missense (n = 5)	44.0 ± 11.7	49.3 ± 9.7	3	4	2	2	1
Non-Cys missense (n = 13)	48.7 ± 12.0	53.9 ± 12.6	7	9	3	6	0
Disruptive (n = 5)	35.8 ± 4.09	70.8 ± 18.5*	4	3	2	2	0

Note: The data in the first two columns were analyzed by one-way ANOVA with Bonferroni post-test (*p < 0.05). The data in the rest five columns were analyzed by Fisher test. AR, aortic regurgitation; MVR, mitral valve regurgitation; TVR, tricuspid valve regurgitation; BAV, bicuspid aortic valve., between the disruptive mutation group and non-Cys missense mutation group.

**Table 4:** Comparison of clinical parameters of patients with and without *FBN1* mutations.

**Mutation Type**	**Age (y) at diagnosis**	**Maximum aortic diameter (mm)**	**Aorticdissection(n=)**	**AR(n=)**	**MVR(n=)**	**TVR(n=)**	**BAV(n=)**	**Patients with hypertension(n=)**
*FBN1*-positive (n = 23)	44.9 ± 11.6	57.0 ± 15.4	14	16	7	10	1	7 (30.4%)
*FBN1*-negative (n = 123)	51.6 ± 13.6*	54.4 ± 12.9	78	75	31	23	10	77 (62.6%)*

Note: AR, aortic regurgitation; MVR, mitral valve regurgitation; TVR, tricuspid valve regurgitation; BAV, bicuspid aortic valve. Data in the first two columns were analyzed by Student t test. Data in the rest of six columns were analyzed by Chi-square test. *p < 0.05.
