# Supplementary material for: Wide mutation spectrum and frequent variant Ala27Thr of FBN1 identified in a large cohort of Chinese patients with sporadic TAAD
Source: Sci Rep. 2015 Aug 14;5:13115. doi: 10.1038/srep13115 (PMC4536522; doi:10.1038/srep13115)
Supplement: Supplementary Information [file srep13115-s1.pdf]

Supplementary information

Wide mutation spectrum and frequent variant Ala27Thr of *FBN1* identified in a large cohort of Chinese patients with sporadic TAAD

Jun Guo[1], Lun Cai[1], Lixin Jia[1], Xiaoyan Li[1], Xin Xi[1], Shuai Zheng[1], Xuxia Liu[1], Chunmei Piao[1], Tingting Liu[1], Zhongsheng Sun[2], Tao Cai[1, 2] & Jie Du[1]*

[1] Beijing Anzhen Hospital, Capital Medical University; The Key Laboratory of Remodeling-Related Cardiovascular Diseases, Ministry of Education; Beijing Collaborative Innovation Center for Cardiovascular Disorders; Beijing Institute of Heart, Lung & Blood Vessel Disease, Beijing, China.

[2] The Institute of Genomic Medicine, Wenzhou Medical College, Wenzhou, China.

* Correspondence to: Jie Du, Beijing Institute of Heart, Lung, and Blood Vessel Diseases, Beijing Anzhen Hospital Affiliated to the Capital Medical University, Beijing 100029, China.

E-mail: jdu@bcm.edu

Supplementary figures

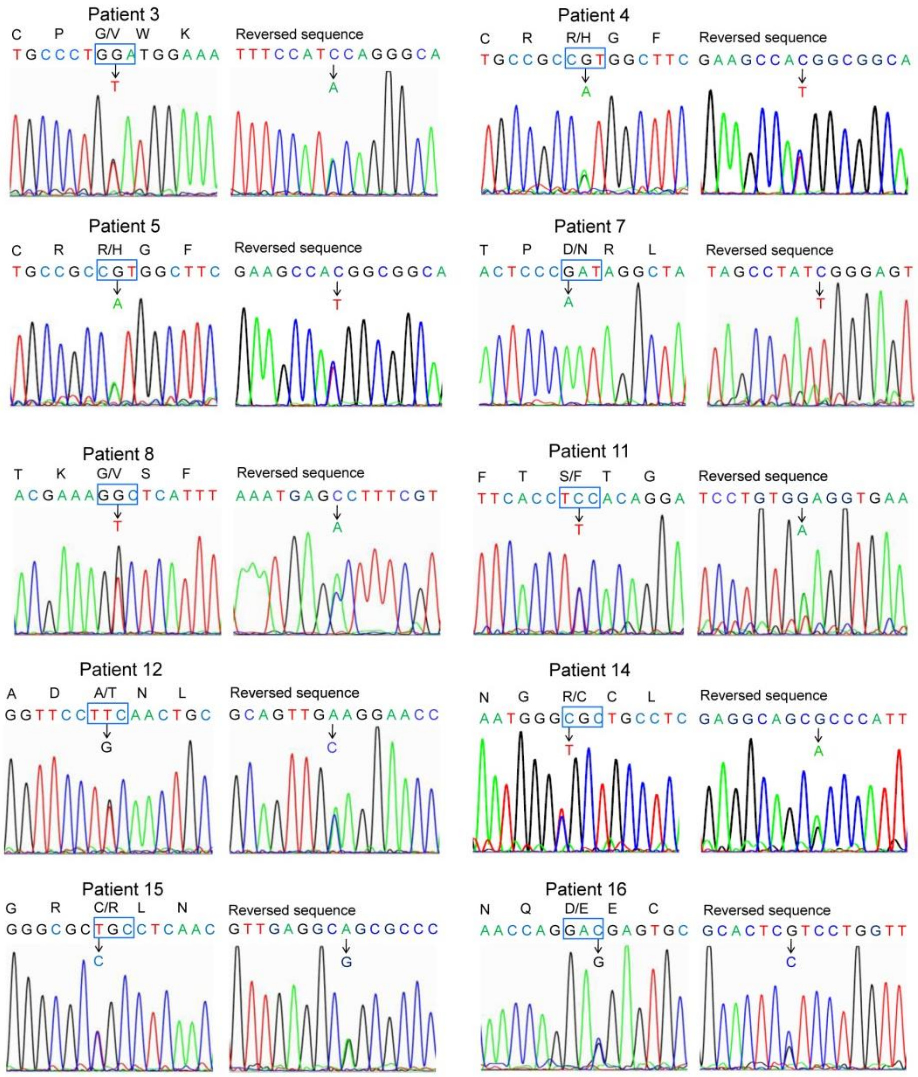

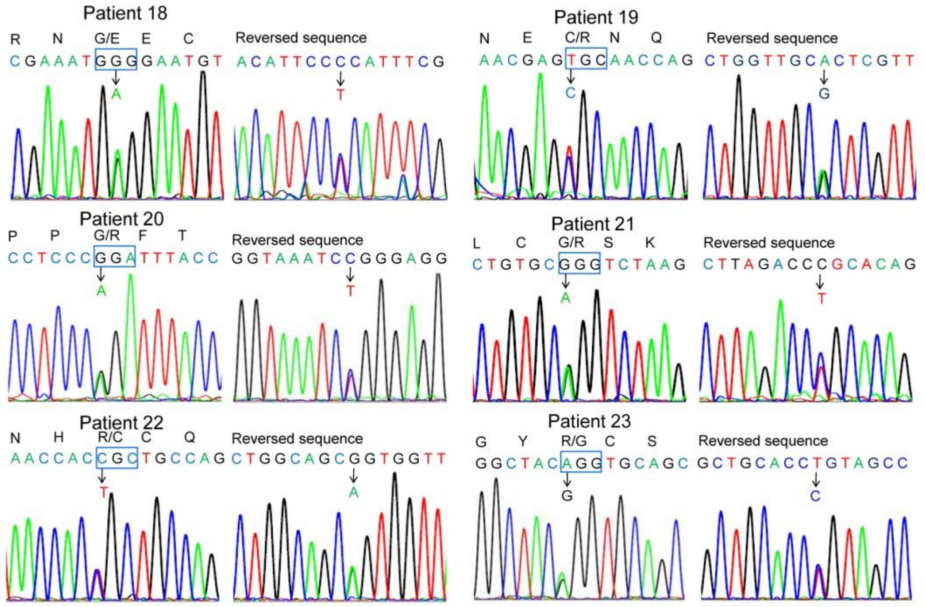

Figure S1 | Sanger sequencing chromatograms in both directions show 16 missense mutations. Clinical conditions and detailed genotypes are presented in Table 1. In addition, the c.79G>A (p.Ala27Thr) mutation in Patient 1 and 2 is shown separately in Fig. 2c. Five disruptive mutations are shown in Fig. 3. The panel is in order from Patient 3 to Patient 23.

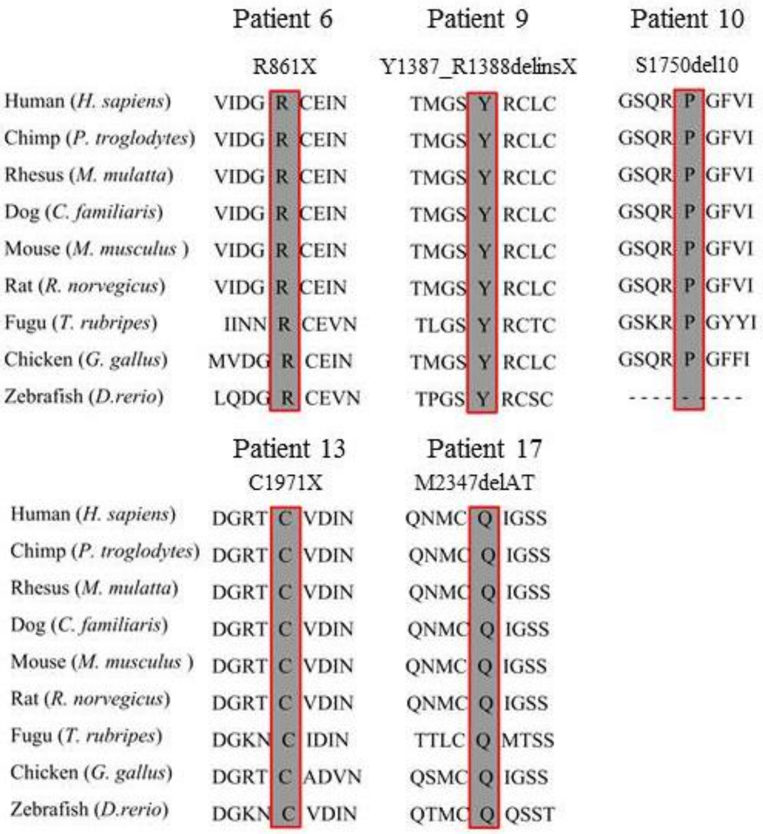

Figure S2｜Multiple sequence alignments show the evolutionary conservation of the boxed residues that are mutated and are predicted to be loss-of-function in five patients. Clinical conditions and detailed genotypes are presented in Table 1. The panel is in order from the residue position R861 to M2347.

	Patient 3<br>G70V	Patient 4 & 5<br>R233H	Patient 7<br>D1191N	Patient 8<br>G1301V
Human (*H. sapiens*)	YCCP G WKTL	HPCR R GFIP	HSTP D RLFC	ENTK G SFIC
Chimp (*P. troglodytes*)	YCCP G WKTL	HPCR R GFIP	HSTP D RLFC	ENTK G SFIC
Rhesus (*M. mulatta*)	YCCP G WKTL	HPCR R GFIP	HSTP D RLFC	ENTK G SFIC
Dog (*C. familiaris*)	YCCP G WKTL	HPCR R GFIP	HSTP D RLFC	ENTK G SFIC
Mouse (*M. musculus*)	YCCP G WKTL	HPCR R GFIP	HPTH D RLFC	ENTK G SFIC
Rat (*R. norvegicus*)	YCCP G WKTL	HPCR R GFIP	HPTH D RLFC	ENTK G SFIC
Fugu (*T. rubripes*)	YCCP G WKTL	NPCR R GFIP	QATP D RQSC	ENTK G SFIC
Chicken (*G. gallus*)	YCCP G WKTL	HPCR R GFIP	QSTA D KLHC	ENTK G SFIC
Zebrafish (*D.rerio*)	YCCP G WKTL	LPCT R GFIP	QTTP D WKGC	ENTK G SFIC

	Patient 11<br>S1843F	Patient 12<br>F1912C	Patient 14<br>R2306C	Patient 15<br>C2307R
Human (*H. sapiens*)	YRFT S TGQC	TIGS F NCRC	CENG R CLNT	ENGR C LNTR
Chimp (*P. troglodytes*	YRFT S TGQC	TIGS F NCRC	CENG R CLNT	ENGR C LNTR
Rhesus (*M. mulatta*)	YRFT S TGQC	TIGS F NCRC	CENG R CLNT	ENGR C LNTR
Dog (*C. familiaris*)	YRFT S TGQC	TIGS F NCRC	CENG R CLNT	ENGR C LNTR
Mouse (*M. musculus*)	YRLT S TGQC	TIGS F NCRC	CENG R CLNT	ENGR C LNTL
Rat (*R. norvegicus*)	YRLT S TGQC	TIGS F NCRC	CENG R CLNT	ENGR C LNTL
Fugu (*T. rubripes*)	YRFT P TGQC	TVGS Y NCLC	CKNG R CINT	KNGR C INTV
Chicken (*G. gallus*)	YRFT S TGRC	TIGS F NCRC	CENG R CVNT	ENGR C VNTV
Zebrafish (*D.rerio*)	YRRA C IGES	TVGS Y NCLC	CKNG R CVNT	KNGR C VNTV

	Patient 16<br>D2329E	Patient 18<br>G2416E	Patient 19<br>C2448R	Patient 20<br>G2514R
Human (*H. sapiens*)	SPNQ D ECLD	VCRN G ECVN	DLNE C NQAP	KCPP G FTQH
Chimp (*P. troglodytes*	SPNQ D ECLD	VCRN G ECVN	DLNE C NQAP	KCPP G FTQH
Rhesus (*M. mulatta*)	SPNQ D ECLD	VCRN G ECVN	DLNE C NQAP	KCPP G FTQH
Dog (*C. familiaris*)	SPAQ D ECLD	VCRN G ECVN	DLNE C NQAP	KCPP G FTQH
Mouse (*M. musculus*)	SPTQ D ECLD	VCRN G ECVN	DLNE C NQAP	KCPP G FTQH
Rat (*R. norvegicus*)	SPTQ D ECLD	VCRN G ECVN	DLNE C NQAP	KCPP G FTQH
Fugu (*T. rubripes*)	SSTA T ECID	LCKN G QCFN	DVDE C IQAP	KCPP G FSQH
Chicken (*G. gallus*)	SDTQ N ECLD	VCRN G ECIN	DLNE C NESP	KCPP G FTQH
Zebrafish (*D.rerio*)	-RNR H ECID	LWHS G - FEK	NMDE C SQSP	KCPS G FTQH

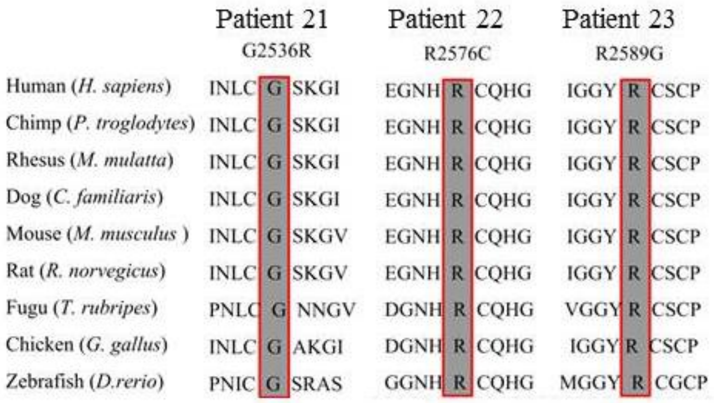

Figure S3 | Multiple sequence alignments show the evolutionary conservation of the boxed residues that are substituted as missense mutations in 16 patients. The A27T is shown in a separate figure (Fig. 2). Clinical conditions and detailed genotypes are presented in Table 1. The panel is in order from the residue position G70 to R2589.

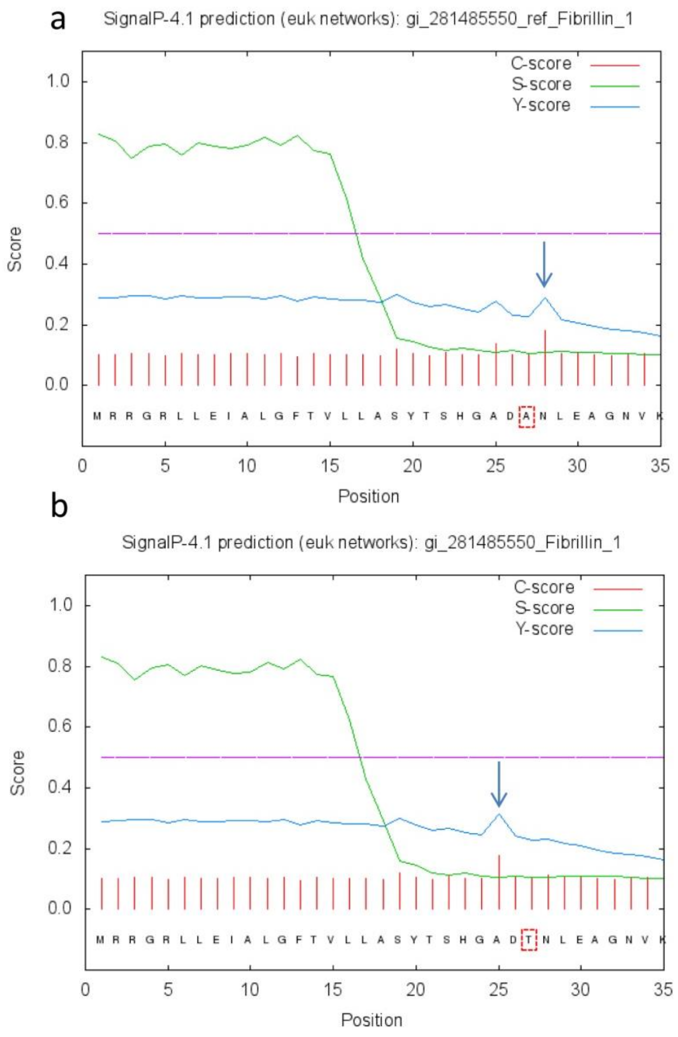

Figure S4丨Comparison of the signal peptide sequence of the wild-type (Ala27) (a) and mutant (Thr27) (b) by SignalP. The software yields three parameters: 1) C-score is the cleavage score at every amino acid position; 2) S-score is the signal peptide prediction for every amino acid position, with a high score indicating that the particular amino acid is part of a functional signal peptide; 3) the Y-score is a derivative of the C-score combined with the S-score, resulting in a better cleavage site prediction.

Supplementary tables

Supplementary Table S1 | Summary statistics for targeted NGS of 23 patients with *FBN1* mutation

Sample	Aligned	Average sequencing depth on target	Coverage of target region	Fraction of target covered with at least 4x	Fraction of target covered with at least 10x	Fraction of target covered with at least 20x
1	93.04%	47.19	79.30%	76.00%	75.10%	73.40%
2	94.51%	56.74	85.60%	75.80%	74.90%	73.80%
3	94.98%	51.85	77.00%	75.70%	75.10%	74.00%
4	93.77%	43.64	94.50%	78.20%	75.20%	74.10%
5	94.20%	58.65	89.00%	76.60%	74.90%	73.80%
6	93.51%	52.01	78.00%	75.90%	74.60%	73.80%
7	95.26%	55.28	79.10%	76.10%	75.30%	73.90%
8	94.10%	50.87	82.60%	75.90%	75.30%	74.50%
9	93.95%	50.31	78.00%	76.00%	74.70%	73.70%
10	94.80%	49.24	79.60%	75.90%	74.80%	74.00%
11	95.78%	62.38	77.80%	76.60%	75.20%	74.20%
12	95.47%	69.67	79.30%	76.40%	75.30%	74.40%
13	93.38%	46.64	77.60%	76.90%	74.90%	73.80%
14	95.28%	47.96	76.80%	76.30%	74.80%	74.10%
15	93.33%	48.76	85.90%	76.40%	75.00%	73.80%

16	95.43%	53.66	78.90%	75.90%	75.00%	74.00%
17	95.04%	55.33	79.60%	76.00%	75.10%	74.00%
18	94.93%	58.87	80.40%	75.40%	74.70%	73.70%
19	95.18%	54.34	79.20%	75.40%	74.90%	73.90%
20	91.20%	44.14	76.40%	75.30%	74.60%	73.00%
21	94.64%	43.61	78.30%	75.50%	74.70%	73.50%
22	95.07%	51.00	80.00%	76.00%	75.50%	74.30%
23	95.21%	50.11	79.70%	75.50%	75.10%	74.40%
Average	94.44%	52.27	80.55%	76.07%	74.99%	73.92%

Supplementary Table S2 | Comparison between the reported *FBN1* mutations and the present study

#	Age (yr)	Sex	Cardiovascular	Skeletal	Familial history	Nucleotide change	Protein change	Ref
6	35	M	Maj	-	-	2581C>T	Arg861X	This report
	43	M	Maj	Min	-	2581C>T	Arg861X	[s1]
19	59	M	Maj	-	-	7342T>C	Cys2448Arg	This report
	13	M	Min	Maj	-	7342T>C	Cys2448Arg	[s2]

Note: Maj, major involvement; Min, minor involvement; -, absent. No abnormalities were observed in ocular, pulmonary, or skin in either groups.

Supplementary Table S3 | *FBN1* mutation analysis in 5 unrelated patients with MFS

#	Sex	Age (y)	Nucleotide Change	Protein change	Module	Cardiovascular system	Other system
24	F	56	c.332G>A	p.Cys111Tyr	cb EGF-like 1	TAA, AR	ARA
25	F	24	c.978_979delCA	p.Thr326delCA	cb EGF-like 2	Dissec, TAA, AR	SC; SM; PNX
26	M	16	c.1876delC	p.Gly626delC	cb EGF-like 6	Dissec, AR	ARA
27	F	24	c.2562G>A	p.Trp854X	hybrid motif 2	Dissec, AR, TVR	ARA
28	M	61	c.5431G>A	p.Glu1811Lys	cb EGF-like 26	TAA	SC; CA

Note: MFS, Marfan Syndrome; TAA, thoracic aortic aneurysms; Dissec, thoracic aortic dissection; AR, aortic regurgitation; TVR, tricuspid valve regurgitation; ARA, arachnodactyly; SC, scoliosis; SM, severe myopia due to ectopia lentis; PNX, spontaneous pneumothorax; CA, carinatum.

Supplementary Table S4 | List of all probes used to enrich for 65 exons of *FBN1*

Start position	End position	Start position	End position
48703147	48703281	48766427	48766598
48703284	48703584	48766696	48766857
48704721	48704938	48773802	48774013
48707694	48707980	48775969	48776179
48712868	48713041	48777525	48777713
48713739	48713922	48779252	48779427
48714101	48714305	48779493	48779662
48717531	48717708	48780265	48780481
48717886	48718091	48780540	48780728
48719715	48719996	48781999	48782292
48720497	48720705	48784610	48784783
48722821	48722995	48786336	48786483
48725016	48725217	48787273	48787496
48726752	48726944	48787621	48787792
48729113	48729307	48788249	48788440
48729464	48729625	48789416	48789603
48729917	48730134	48791131	48791273
48733872	48734071	48795948	48796125
48736690	48736893	48797172	48797367

48737554	48737735	48800730	48800934
48738855	48739052	48802216	48802394
48740940	48741129	48805699	48805896
48744722	48744911	48807535	48807757
48748787	48748978	48808365	48808583
48752392	48752537	48812806	48813036
48755232	48755471	48818282	48818492
48756047	48756251	48826232	48826435
48757727	48757906	48829768	48830037
48757935	48758088	48888435	48888599
48760095	48760317	48892289	48892449
48760599	48760769	48902878	48903049
48762786	48762982	48905162	48905322
48764698	48764900	48936753	48936982

References

s1. Katzke, S. *et al.* TGGE screening of the entire FBN1 coding sequence in 126 individuals with marfan syndrome and related fibrillinopathies. *Hum Mutat* **20**, 197-208 (2002).

s2. Sakai, H. *et al.* Comprehensive genetic analysis of relevant four genes in 49 patients with Marfan syndrome or Marfan-related phenotypes. *Am J Med Genet A* **140**, 1719-1725 (2006).
